# 25I-NBOH: a new potent serotonin 5-HT_2A_ receptor agonist identified in blotter paper seizures in Brazil

**DOI:** 10.1007/s11419-017-0357-x

**Published:** 2017-02-16

**Authors:** Luciano Chaves Arantes, Ettore Ferrari Júnior, Luciano Figueiredo de Souza, Andriele Costa Cardoso, Thaynara Lino Fernandes Alcântara, Luciano Morais Lião, Yuri Machado, Rogério Araújo Lordeiro, José Coelho Neto, Ana Flávia B. Andrade

**Affiliations:** 1 Instituto de Criminalística, Polícia Civil Do Distrito Federal, SPO, Lote 23, Bloco E, Brasília, DF 70610-200 Brazil; 2 Instituto de Criminalística Leonardo Rodrigues, Superintendência da Polícia Técnico-Científica do Estado de Goiás, Goiânia, GO 74425-030 Brazil; 3 Instituto de Química, Universidade Federal de Goiás, Campus Samambaia, Goiânia, GO 74001-970 Brazil; 4 Divisão de Laboratório, Instituto de Criminalística da Polícia Civil de Minas Gerais, Rua Juiz de Fora, 400, Belo Horizonte, MG 30180-060 Brazil; 5 Departamento de Física e Química, Pontifícia Universidade Católica de Minas Gerais, Avenida Dom José Gaspar, 500, Belo Horizonte, MG 30535-901 Brazil; 6 School of Chemistry, University of Lincoln, Brayford Pool, Lincoln, LN6 7TS UK

**Keywords:** 25I-NBOH, NBOH, Phenethylamine derivatives, NPS, 5-HT_2A_ receptor agonist

## Abstract

A new potent serotonin 5-HT_2A_ receptor agonist was identified in blotter papers by several state level forensic laboratories in Brazil. The 25I-NBOH is a labile molecule, which fragments into 2C-I when analyzed by routine seized material screening gas chromatography (GC) methods. GC–mass spectrometry (MS), liquid chromatography–quadrupole time-of-flight-MS, and Fourier transform infrared and nuclear magnetic resonance analyses were performed to complete molecular characterization. Individual doses range from 300 to 1000 μg. Despite its being a potent 5-HT_2A_ receptor agonist, 25I-NBOH is neither registered in the United Nations Office on Drugs and Crime (UNODC) nor classified as a scheduled substance in most countries. Sweden and Brazil seem to be the only countries to control 25I-NBOH. To our knowledge, this is the first scientific report dealing with identification of 25I-NBOH in actual seizures.

## Introduction

The global market for new psychoactive substances (NPSs) continues to expand, and seizures have increased 18% since 2014 [1]. The number of different drugs available is a striking feature of this market. Just in 2015, 75 new substances were reported [2]. Phenethylamines have a stable presence in the drug market, being reported every year since its first report in 2008. Many reported phenethylamines are actually derivatives of previously reported substances such as the series of 2,5-dimethoxy ring-substituted phenethylamines (2C series) [1].

**Fig. 1:**
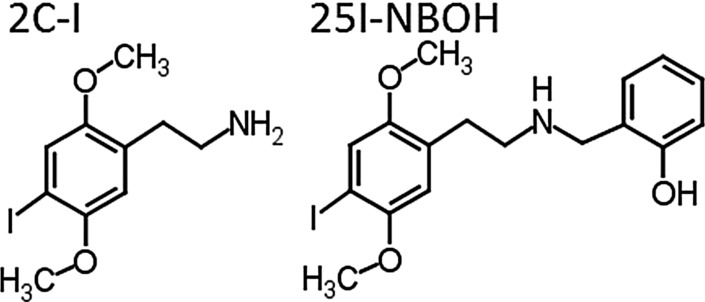
Chemical structures of 2C-I and 25I-NBOH

Starting from 2014, shortly after NBOMes were prohibited in Brazil, forensic laboratories around the country started to receive blotter papers and detected the presence of 2C-I by gas chromatography–mass spectrometry (GC–MS), which was considered as a golden standard for this type of sample. Quantification analyses, however, demonstrated that drug concentrations present in these samples were much below the minimal dosage reported to be able to produce any biological effect in the human body. This fact instigated a more detailed analysis of the samples by liquid chromatography–quadrupole time-of-flight-mass spectrometry (LC–QTOF-MS), which led us to identify 25I-NBOH (2-({[2-(4-iodo-2,5-dimethoxyphenyl)ethyl]amino}methyl)phenol) as the real compound present in the analyzed samples (Fig. 1). To complete structural characterization of this compound, collaboration with other forensic laboratories was established, and nuclear magnetic resonance (NMR) and Fourier transform infrared (FTIR) analyses were also carried out [3].

The NBOH compounds are *N*-benzylhydroxy derivatives of the 2C hallucinogen family often containing a halogen group (i.e., chlorine, bromine, or iodine) at position 4 of the A ring and a hydroxyl group in the position 2′ in the B ring. The NBOH compounds are proposed as a legal alternative for the NBOMe compounds, even if their physiological and toxicological properties are not fully reported. The *N*-benzyl, more specifically *N*-(2-methoxy)benzyl phenethylamine substituents, dramatically improved both binding affinity and functional activity, resulting in vivo 5-HT_2A_ activation. These compounds including 25I-NBOH are truly selective, high affinity, potent, and highly efficacious agonists of the human 5HT_2A_ receptor, responsible for subjective and behavioral effects [4].

Scientific data regarding NBOH compounds are very scarce. Only a few pharmacological studies on mice or computational models have been conducted on these drugs [4–10]. Only a single paper reported the recovery of 25C-NBOH, together with 25C-NBOMe and 2C-C from urine samples in three adult males, one of whom died experiencing respiratory difficulties. Internally, the autopsy showed mild to moderate coronary atherosclerosis, biventricular dilatation, mild right ventricular hypertrophy and bilateral pulmonary edema and congestion [11].

## Materials and methods

### Chemical and reagents

Methanol with 0.1% formic acid CHROMASOLV^®^ for high-performance liquid chromatography (HPLC), ≥99.9%, and water with 0.1% formic acid CHROMASOLV^®^ for HPLC, were purchased from Sigma-Aldrich (St. Louis, MO, USA). Methanol, the LC–MS grade, ≥99.9%, was purchased from Scharlau (Barcelona, Spain). Deuterated chloroform (CDCl_3_) with 99.8% isotopic purity, containing 0.03% (v/v) tetramethylsilane (TMS), was obtained from Sigma-Aldrich. A certified reference standard of 25I-NBOH was purchased from Cayman Chemical, Ann Arbor, MI, USA. A 2C-I certified reference standard was kindly donated by the United Nations Office on Drugs and Crime (UNODC).

### Samples

**Fig. 2:**
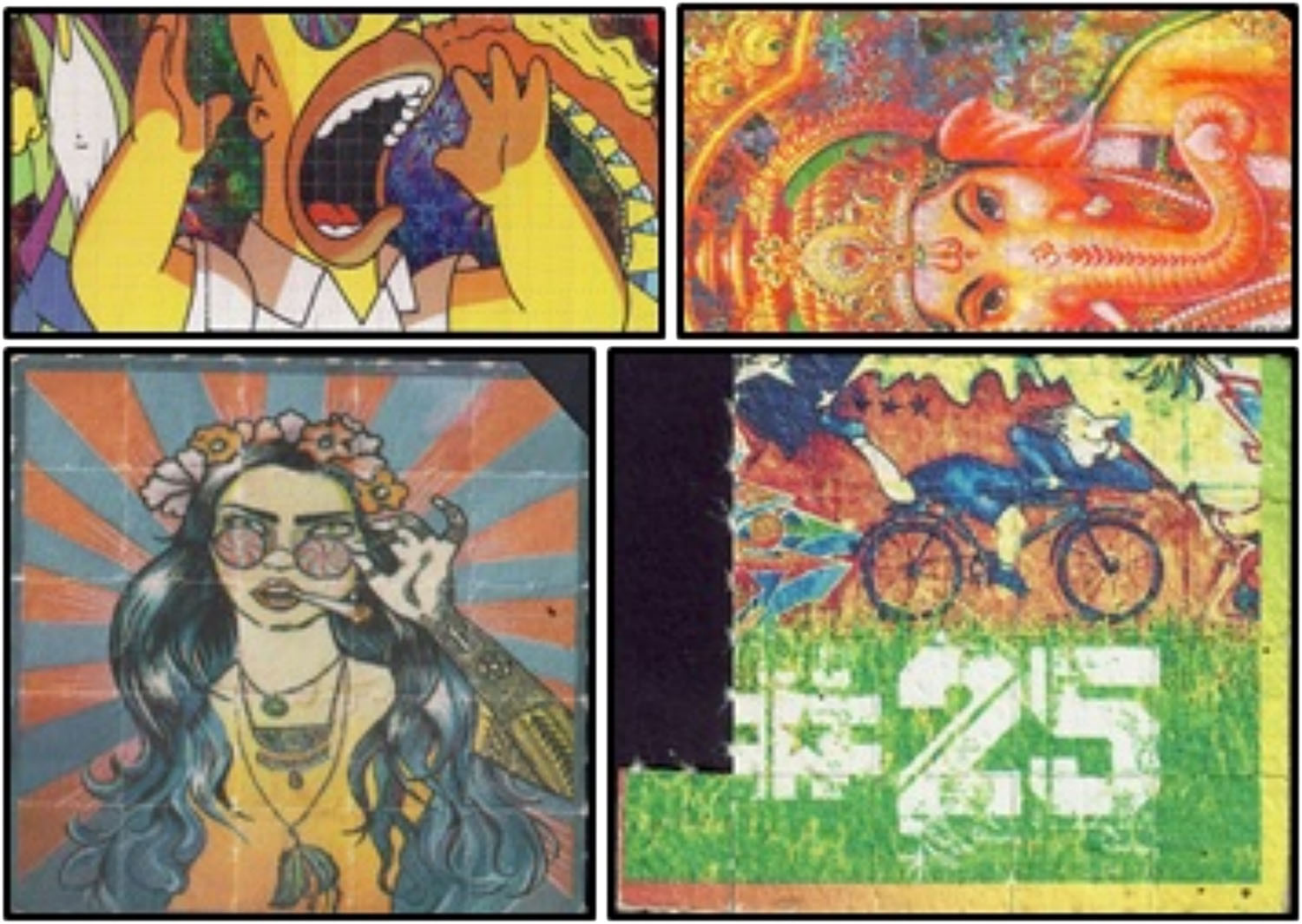
Blotter papers containing illicit substances

Blotter papers suspected to contain illicit substances were seized between 2014 and 2016 by police forces from the Federal District, Goiás State and Minas Gerais State. These blotters usually represented a colorful side including drawings of famous celebrities, cartoon characters, fruits, etc. (Fig. 2) and a white side. Sometimes on the white side a chemical structure is displayed.

### Instruments

GC–MS analyses were performed using a combined Agilent 7890A gas chromatograph and 5975C mass spectrometer (Agilent Technologies, Santa Clara, CA, USA). The system was controlled with Agilent Chemstation GC/MS Software version E 02.02.1431. LC–TOF-MS analysis was performed with an Agilent 6540 QTOF mass spectrometer equipped with a Jet Stream interface for electrospray ionization (ESI), with an Agilent 1290 Infinity LC instrument. Compounds were detected and reported from accurate mass scan data using an Agilent MassHunter Qualitative Analysis B 06.00 and Personal Compound Database and Library (PCDL) B 02.00 software, and the quantitative analysis was performed using an Agilent MassHunter Quantitative B 07.01. All NMR data were acquired at 25 °C using a Bruker Avance III 500 spectrometer (Bruker, Karlsruhe, Germany) operated at 11.75 T (^1^H resonance frequency 500.13 MHz), equipped with a 5 mm triple resonance broadband. All acquisition and processing were conducted by TopSpin software (Version 3.2 Bruker BioSpin).

Attenuated total reflectance (ATR)-FTIR spectra were taken using a Nicolet™ *iZ*10 spectrometer equipped with an EverGlo IR source, DLaTGS room temperature infrared (IR) detector and single-bounce Smart Orbit™ accessory module with a diamond ATR crystal. All hardware was purchased from Thermo Fischer Scientific Inc. (Waltham, MA, USA).

## Methods

For GC–MS and LC–QTOF-MS analyses, extraction of blotter paper samples was carried out with no pretreatment at room temperature as follows. One blotter was placed in a test tube, and 10 mL of methanol was added followed by sonication in an ultrasound bath for 10 min. Ten microliters of this solution was then added to a vial containing 1 mL of methanol, and GC–MS and/or LC–QTOF-MS analyses were performed.

The GC was equipped with an Agilent J&W DB-1 ms fused-silica capillary GC column (30 m × 0.25 mm i.d., 0.25 μm film thickness). The sample injection volume was 0.5 µL at a split ratio of 50:1. Helium was used as a carrier gas at 1 mL/min. The inlet line temperature was set at 280 °C, and the injector temperature was maintained at 200 °C. The GC oven temperature program started with an initial temperature of 100 °C and initial hold time of 0 min. Then temperature increased to 300 °C at a rate of 6 °C/min. The maximum temperature was held for 5.67 min. An electron ionization source was utilized with a mass analyzer operating in full scan mode (*m/z* 34–600).

The LC–QTOF-MS method was performed as follows. For internal mass calibration, two reference masses were infused to perform mass correction in every scan: 121.050873 (purine) and 922.009798 (HP-0921) in positive ionization mode. Mobile phase A consisted of 0.1% formic acid in water, and the mobile phase B consisted of 0.1% formic acid in methanol. Separation was performed with a Zorbax Eclipse Plus C18 (100 × 2.1 mm i.d., particle size 1.8 μm) column (Agilent Technologies). Injection volume was 1 μL. The instrument acquired data using the following parameters: drying gas temperature, 350 °C; drying gas flow, 8.0 L/min; nebulizer, 35 psi; sheath gas temperature, 400 °C; sheath gas flow, 11 L/min; VCap, 3500 V; nozzle, 0 V; skimmer, 65 V; and octopole RF peak, 750 V.

The ATR-FTIR spectra were collected directly from blotter papers. Two spectra were collected from each blotter, one at the back side, which normally presented no artwork, and one at the front side, where the main artwork was located.

Each spectrum were averaged over 16 scans, taken at 4 cm^−1^ resolution; maximum detector window aperture and minimum interferometer mirror speed were set in the range of 400–4000 cm^−1^. Background signal was averaged over eight scans prior to each measurement.

For NMR analysis, five blotters were placed in a 2 mL vial and 600 µL of deuterated chloroform (CDCl_3_) was added followed by sonication in an ultrasound bath for 5 min. NMR spectra were recorded using a *zg* sequence. One hundred twenty-eight (128) free induction decays (FIDs) were collected into 64 k data points using a 9.49 µs pulse width (90° pulse angle), spectral width of 10,000 Hz, relaxation delay of 1.0 s and an acquisition time of 3.28 s. The phase and baseline were automatically corrected. An exponential weighing factor corresponding to line broadening of 0.3 Hz was applied to the FIDs. The heteronuclear single quantum coherence (HSQC) and heteronuclear multiple-bond correlation (HMBC) experiments were acquired with spectral windows of 10,000 and 37,731.109 Hz for ^1^H and ^13^C, respectively.

### Results and discussion

The GC–MS analysis of blotter papers seized by several state level forensic laboratories in Brazil indicated the presence of 2C-I. Retention time (RT) and fragmentation patterns were confirmed with the 2C-I standard. Quantification analysis demonstrated an average of 2 mg/mL in each sample. The known doses for 2C-I that have any hallucinogen effect in human body are above 14 mg [12], which led us to perform further analysis.

Preliminary LC–QTOF-MS analysis using fragmentor voltage at 110 V in the scan mode showed a total ion current chromatogram of the target compound with a peak at 8.519 min. However, the ESI mass spectrum showed two exact mass fragments: *m/z* 308.0142 [M + H]^+^ (2C-I) and *m/z* 414.0561 [M + H]^+^. A search in our PCDL (Agilent Technologies) was carried out and suggested that the ion at *m/z* 414.0561, compatible with the molecular formula of C_17_H_20_INO_3_, represented the compound 2-({[2-(4-iodo-2,5-dimethoxyphenyl)ethyl]amino}methyl) phenol (25I-NBOH). The error between the observed and theoretical mass number of [M + H]^+^ was less than 2 ppm. Therefore, analytical characterization of this new compound was necessary.

**Fig. 3:**
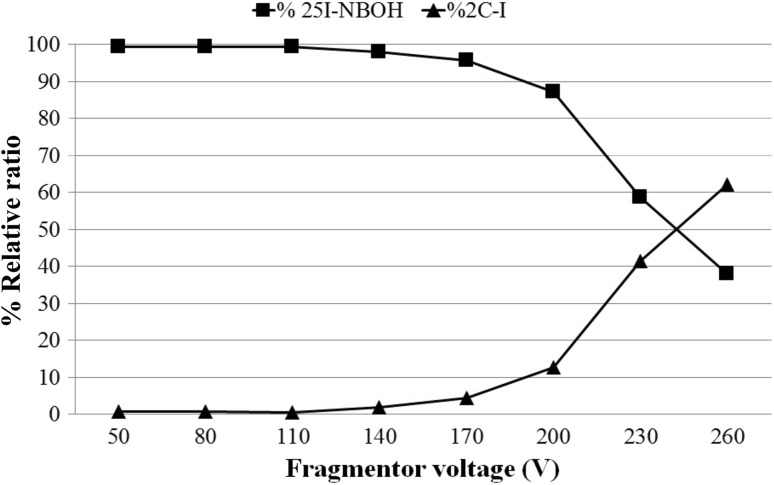
Cleavage of 25I-NBOH using different fragmentor voltages. Percent relative volume = percentage of 2C-I and 25I-NBOH in relation to the sum of both peaks in the same retention time

The collision induced dissociation (CID) was carried out in order to identify the precursor ion [M + H]^+^ present in the analyzed sample. Considering that at higher fragmentor voltage settings, fragmentation can be initiated in the region between the end of the transfer capillary and the first skimmer cone, high-resolution mass data was acquired using different fragmentor voltages (50–260 V). Low energy spectra predominantly showed the *m/z* 414.0561 (25I-NBOH) ion. The relative intensity of the ions at *m/z* 414.0561 and 308.0142 present an inverse relation. For fragmentor voltages up to 110 V, no *m/z* 308.0142 (2C-I) ion was present. This result indicates that the 2C-I ion previously observed was resulting from 25I-NBOH fragmentation (Fig. 3).

**Fig. 4:**
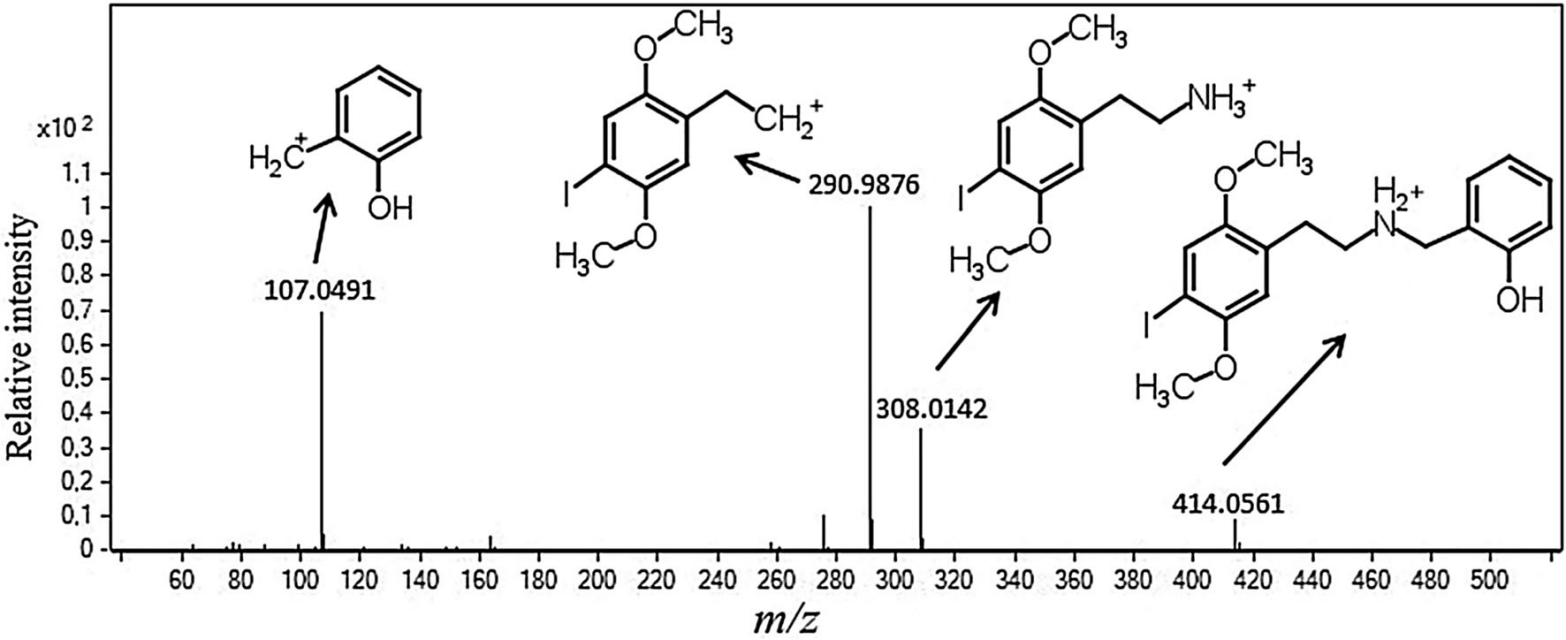
Product ion spectrum of 25I-NBOH showing the corresponding chemical structure at 80 and 20 V of fragmentor voltage and collision energy, respectively, obtained by liquid chromatography–quadrupole time-of-flight-tandem mass spectrometry

Subsequently, the CID analysis was carried out with fixed fragmentor voltage (80 V) and different collision energies (10, 20 and 40 V). The CID spectrum observed at 20 V of collision energy presented the protonated molecule [M + H]^+^ at *m/z* 414.0561 (11), *m/z* 107.0491 (69), *m/z* 308.0142 (40) and *m/z* 290.9876 (100) (Fig. 4). The ions detected at *m/z* 107.0491 and 308.0142 are produced by the cleavage of the C–N bond corresponding to o-cresol and 2C-I, respectively. The ion recorded at *m/z* 290.9876 is formed after the loss of the amine from the 2C-I fragment [9], and it corresponds to the most abundant ion in the CID spectrum of 25I-NBOH. The presented results suggest that 2C-I (*m/z* 308.0142) is a fragment of the 25I-NBOH, explaining the presence of this compound in the high-energy production spectra of this drug.

**Fig. 5:**
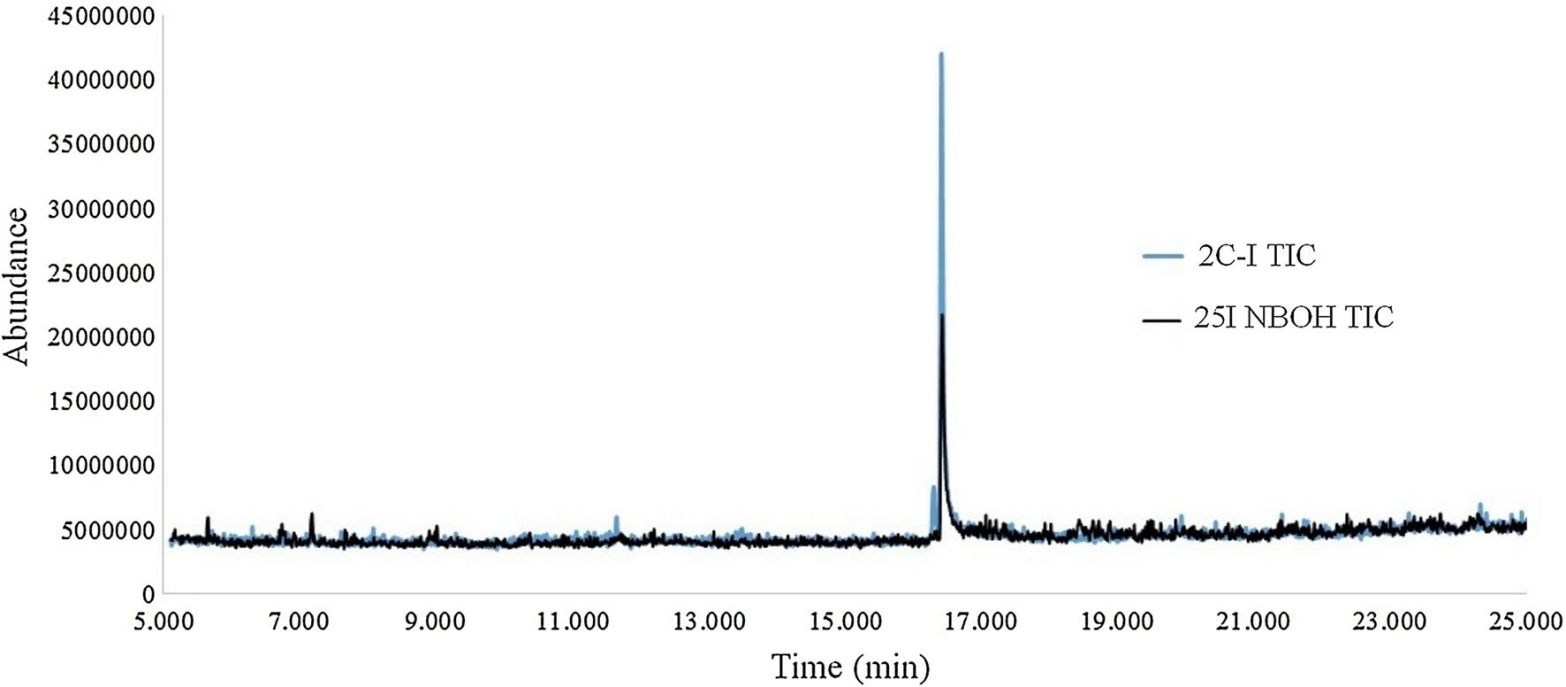
Total ion current chromatogram (TIC) for 25I-NBOH and 2C-I showing the same retention time by gas chromatography–mass spectrometry analyses

GC–MS analyses of the 25I-NBOH reference standard presented the same retention time and fragmentation into 2C-I, being indistinguishable by this technique (Fig. 5). This fact associated with the LC–QTOF-MS results presents fundamental evidence of the instability of the 25I-NBOH to be degraded into 2C-I by the usual high temperature in the CG–MS injector or even with the routine fragmentor voltage by LC–QTOF-MS.

**Fig. 6:**
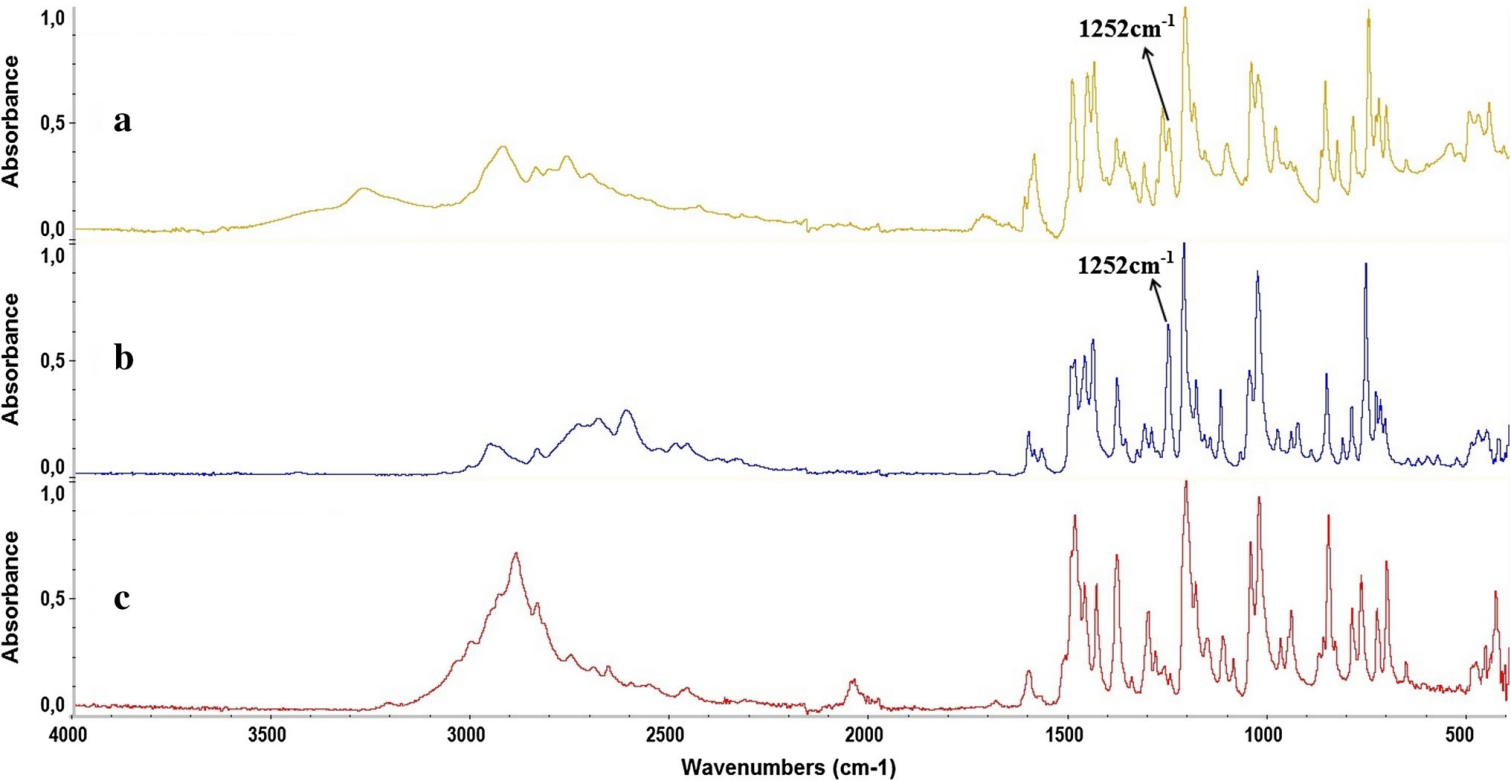
Spectral comparison showing infrared (IR) profiles obtained from the backside of a sample blotter and reference standard spectra. *Arrow* shows the decrease indicating the substitution of OCH_3_ by OH in the 25I-NBOH. **a** Spectral profile from the back side of a blotter containing 25I-NBOH. **b** IR spectrum of 25I-NBOMe (from SWGDRUG IR library). **c** IR spectrum from 2C-I (from SWGDRUG IR library)

ATR-FTIR spectra of the seized blotters were analyzed employing both single and multicomponent IR spectral profile analyses [13] identifying signals matching primarily 25I-NBOMe (SWGDRUG infrared library). Comparative spectrum analyses between the results obtained from blotter papers and references standards for 25I-NBOME and 2C-I were performed, because GC–MS analyses presented a positive result for 2C-I. Interpretation of the ATR-FTIR spectra indicated that, despite the higher degree of similarity with 25I-NBOMe than with 2C-I, a systematic decrease of the spectral band centered around 1250 cm^−1^, characteristic of asymmetric C–O–C vibrations of NBOMe compounds, was observed. The decrease of this peak indicates the substitution of OCH_3_ by OH in the NBOH structure, resulting in an NBOH compound (Fig. 6).

**Fig. 7:**
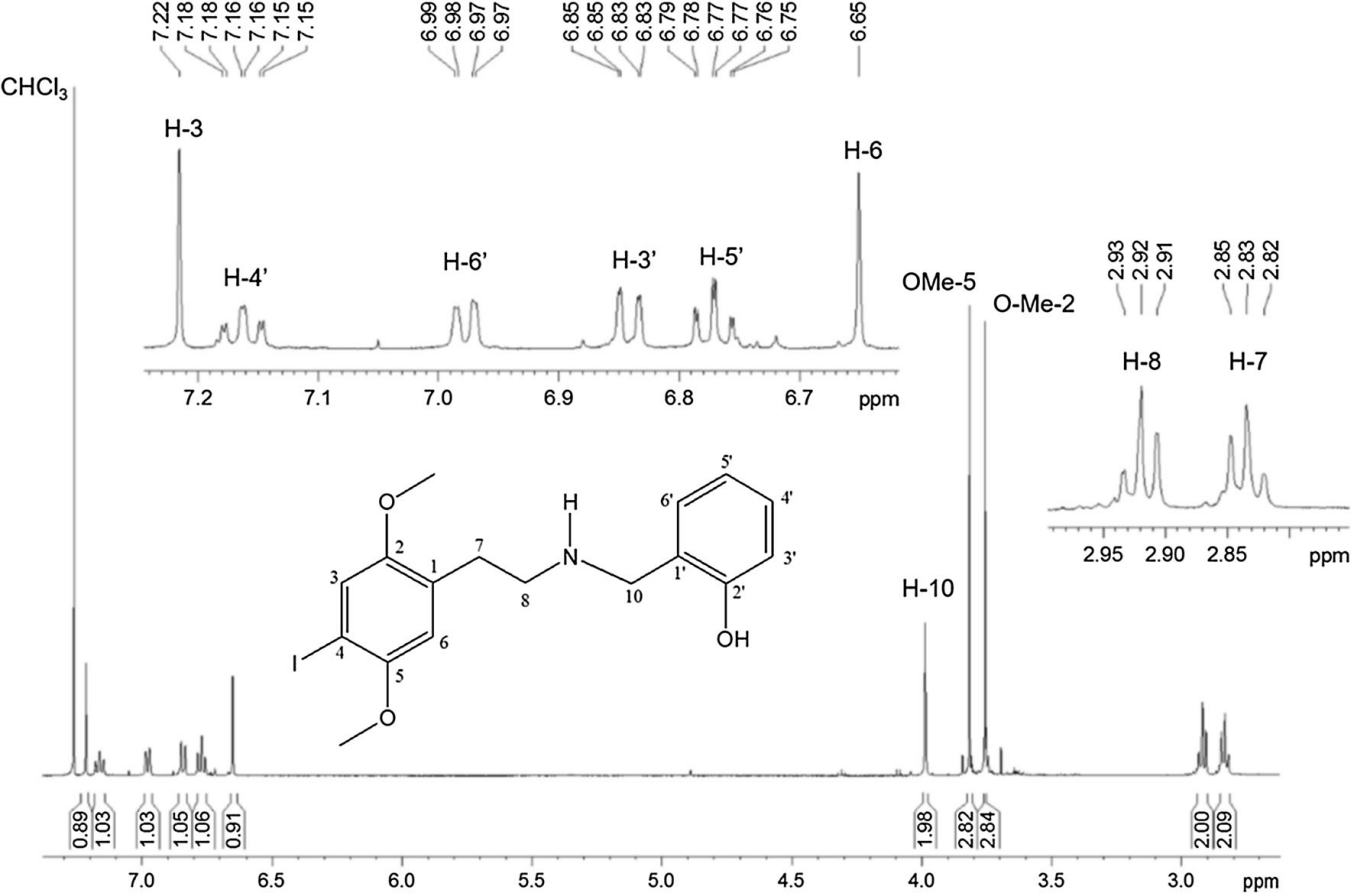
^1^H nuclear magnetic resonance spectra of blotter paper showing the presence of the substance 25I-NBOH

The ^1^H spectrum by NMR spectroscopy obtained for blotter papers is shown in Fig. 7. The multiplicities, coupling constants (*J*) and chemical shifts (*δ*) are attributed to one 1,2,4,5-tetrasubstituted aromatic ring containing two methoxy, one iodine and one ethylmethylamine groups. Attached to this amine group was observed one 1,2-disubstituted aromatic ring that also contained one hydroxyl group.

HSQC and HMBC experiments were used to confirm the carbon skeleton connectivity. These experiments were also used for complete carbon chemical shift assignments. The strong shielding observed for the *ipso*-carbon (C-4; *δ* 82.9) confirmed the iodine presence attached directly to aromatic ring. On the other hand, the deshielding of C-2′ (*δ* 158.2) corroborated the presence of a hydroxyl group substituent. The complete ^1^H and ^13^C signals assignment and the correlations ^1^H-^13^C are presented in Table 1.

**Table 1:** ^1^H and ^13^C nuclear magnetic resonance spectral data for 25I-NBOH (500 MHz, CDCl_3_)

Position	*δ* ^1^H^a^	*δ* ^13^C	^2^ *J*	^3^ *J*
1	–	128.5	–	–
2	–	152.2	–	–
3	7.22 (1H, *s*)	121.7	C-2, C-4	C-1
4	–	82.9	–	–
5	–	152.5	–	–
6	6.65 (1H, *s*)	113.6	C-5, C-1	C-4, C-7
7	2.83 (2H, *t, J* = 7.0 Hz)	30.6	C-8, C-1	C-2, C-6
8	2.92 (2H, *t, J* = 7.0 Hz)	48.0	C-7	C-10, C-1
10	3.98 (2H, *s*)	52.2	C-1′	C-8, C-2′, C-6′
1′	–	122.0	–	–
2′	–	158.2	–	–
3′	6.84 (1H, *dd*, *J* = 8.13, 1.07 Hz)	116.3	C-2′	C-1′, C-5′
4′	7.16 (1H, *ddd*, *J* = 8.13, 7.50, 1.70 Hz)	128.7	–	C-2′, C-6′
5′	6.77 (1H, *ddd*, *J* = 7.50, 7.50, 1.07 Hz)	118.9	C-6′	C-1′, C-3′
6′	6.97 (1H, *dd*, *J* = 7.50, 1.70 Hz)	128.2	–	C-10, C-2′, C-4′
OCH_3_-2	3.75 (*s*)	56.0	–	C-2
OCH_3_-5	3.81 (*s*)	57.0	–	C-5

^a^The multiplicity patterns are abbreviated as singlet (*s*), triplet (*t*), double doublet (*dd*) and double double doublet (*ddd*)

## Conclusions

In recent years, new psychoactive substances have rapidly emerged in drug markets, purportedly as “legal” alternatives to internationally controlled drugs. The correct identification of an NPS is a challenge from an analytical point of view due to new drugs being produced with only slight chemical modifications to the existing structures. In this study, we found a new phenethylamine derivative from the NBOMe compounds in blotter papers seized in three different Brazilian states. Accurate LC–QTOF-MS and ATR-FTIR and NMR analyses enabled us to identify this compound as 25I-NBOH.
